# Diverse origins of hepatitis C virus in HIV co-infected men who have sex with men in Hong Kong

**DOI:** 10.1186/s12985-015-0355-8

**Published:** 2015-08-08

**Authors:** Denise P. Chan, Ada W. Lin, Ka Hing Wong, Ngai Sze Wong, Shui Shan Lee

**Affiliations:** Stanley Ho Centre for Emerging Infectious Diseases, The Chinese University of Hong Kong, Hong Kong, China; Centre for Health Protection, Department of Health, Hong Kong, China

**Keywords:** Acute HCV infection, HIV, Men who have sex with men, Sexual transmission

## Abstract

**Background:**

Worldwide, Hepatitis C (HCV) infection has been increasingly recognized in HIV-positive men who have sex with men (MSM). The objective of this study was to characterize the transmission dynamics of acute HCV infection in HIV-positive MSM in Hong Kong using a molecular approach.

**Findings:**

We retrospectively examined 24 HIV-positive MSM with acute HCV infection diagnosed between 2009 and 2014 in Hong Kong. Detection and molecular characterization of HCV was successfully performed in 22 (91.7 %) patients. Genotype 3a was the most prevalent as identified in 14 (63.6 %) MSM, followed by 1a in 4 (18.2 %), 6a in 2 (9.1 %), and 1each (4.5 %) for 1b and 2a. The high prevalence of genotype 3a in MSM was in stark contrast to its rarity among HCV infected injection drug users (IDU) in Hong Kong. Phylogenetic analyses revealed a monophyletic HCV-3a cluster composing of MSM without injection history, and a homologous pair with HCV-6a genotype. There was otherwise no temporal or genetic clustering of the corresponding HIV sequences.

**Conclusions:**

The origin of sexually acquired acute HCV infections in HIV-positive MSM was diverse and not directly linked with local IDU. The transmission dynamics of HIV and HCV infections in MSM in Hong Kong were evidently unrelated.

## Findings

Globally, needle-sharing in injection drug users (IDU) constitutes the main route of hepatitis C virus (HCV) infection. While sexual transmission of HCV is considered relatively inefficient, it has become increasingly recognized among men who have sex with men (MSM). Since the year 2000, epidemics of acute HCV infection among HIV infected (HIV + ve) MSM in Europe, United States, Australia and Asia have been reported [1–4]. Several cohort studies reported a rising incidence of HCV in HIV + ve MSM following the introduction of HAART, with a pronounced escalation after 2002 [5, 6]. In Hong Kong, the main reservoir of HCV is the IDU community with an anti-HCV seroprevalence of over 80 % [7]. HIV infection has so far been uncommon in IDU because of the long history and high coverage of methadone maintenance. In view of the rising number of HCV infections in MSM, yearly screening for HCV was offered to HIV + ve MSM at the territory’s largest HIV clinic which has a caseload of over 2000. To understand the transmission dynamics of acute HCV infection in HIV + ve MSM, a molecular approach was adopted to evaluate the pattern of HIV/HCV co-infection, based on data collected since screening was introduced.

**Fig. 1:**
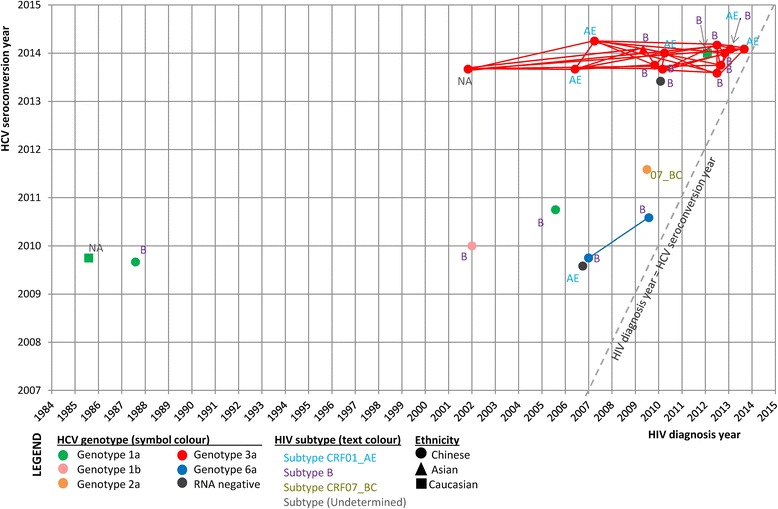
Association between HCV and HIV diagnoses by year and HCV genotypes

This study was approved by Joint Chinese University of Hong Kong – New Territories East Cluster Clinical Research Ethics Committee. Between August 2009 and March 2014, 24 HIV + ve MSM attending the clinic were diagnosed with a recently-acquired HCV infection. The inclusion criteria for the study were anti-HCV seroconversion and/or the detection of positive HCV RNA within the preceding 12 months, in the presence of negative anti-HCV status from previous testing. Some infections were picked up because of deranged liver function and a concurrent diagnosis of sexually transmitted infection (STI) like syphilis, suggesting probable venereal exposure. From the available clinical histories, all infections could be linked to recent episodes of unprotected homosexual contacts. The demographics of HIV/HCV co-infected MSM in our cohort are shown in Table 1. The median age at HCV diagnosis was 32 years (interquartile range [IQR]: 27–41 years). All were ethnic Chinese except 2 Caucasians and 1 non-Chinese Asian, and none gave a history of drug injection. The median interval between HIV diagnosis and HCV seroconversion was 3.1 years (IQR: 1.2–6.5 years). One MSM was diagnosed with HCV infection within 6 months of his HIV diagnosis. The HIV-1 subtype was confirmed in 22 patients: subtype B in 15 (68.2 %), CRF01_AE in 6 (27.3 %) and CRF07_BC in 1 (4.5 %). A majority of the HCV infections diagnosed in these HIV + ve MSM after 2012 belonged to genotype 3a (14/16, 87.5 %) (Fig. 1).

**Table 1:** Characteristics of 24 HIV-positive MSM with sexually acquired acute hepatitis C virus infection in Hong Kong

Characteristics		
Median age at estimated HCV infection, years (IQR)	32	(27–41)
Ethnicity, n (%)		
Chinese	21	(87.5)
non-Chinese	3	(12.5)
Time since HIV infection, years (IQR)	5.0	(2.3–8.2)
On HAART, n (%)	23	(95.8)
Median time from HIV diagnosis to estimated HCV infection, years (IQR)	3.1	(1.2–6.5)
HIV subtype (N = 22)^†^, n (%)		
B	15	(68.2)
CRF01_AE	6	(27.3)
CRF07_BC	1	(4.5)

*HAART* highly active antiretroviral therapy, *HCV* hepatitis C virus, *IQR* interquartile range, *MSM* men who have sex with men

^†^Of those HCV RNA-positive patients

**Fig. 2:**
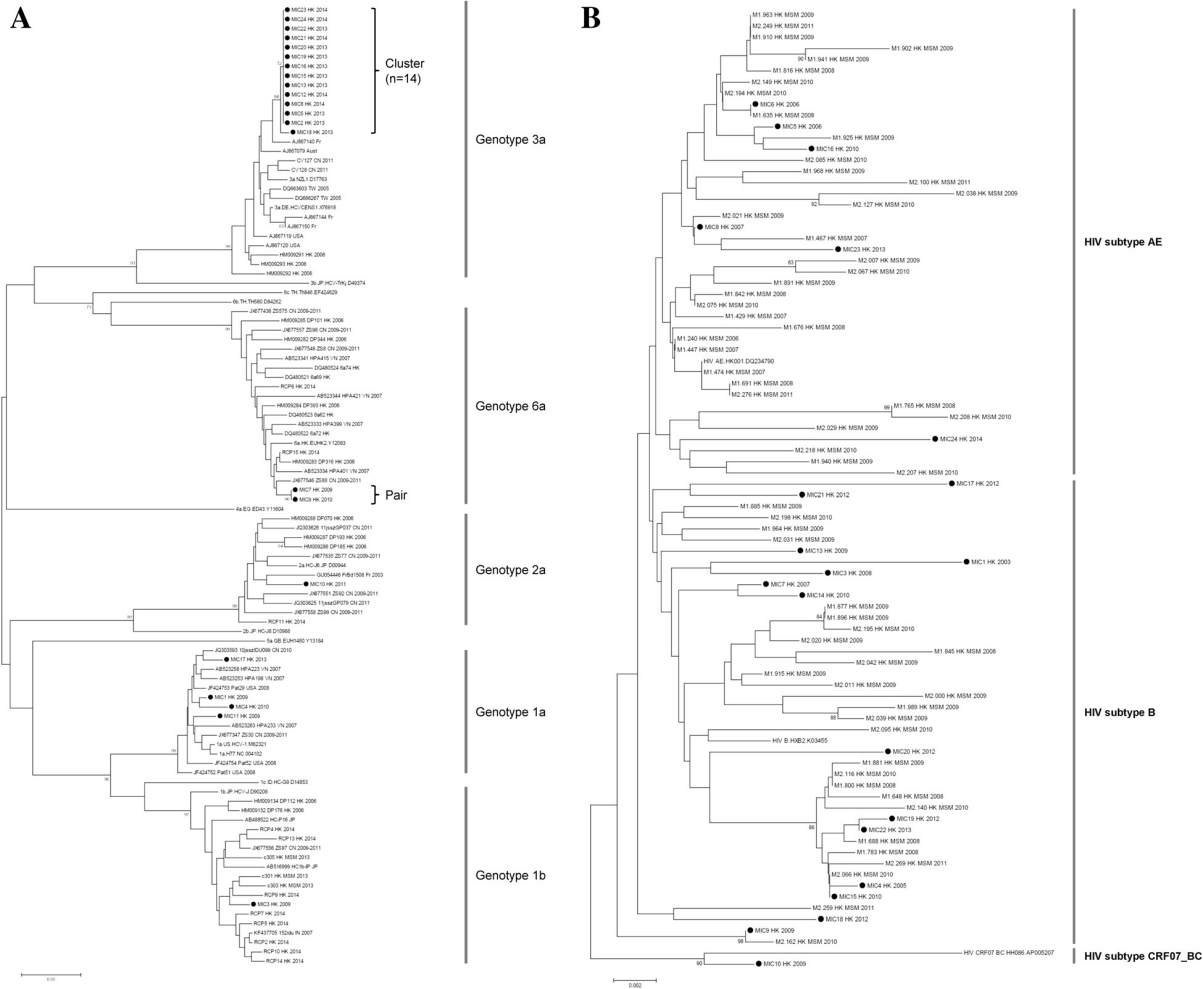
Neighbor-joining phylogenetic trees of HCV (**a**) and HIV (**b**) strains isolated from HIV/HCV co-infected MSM in Hong Kong. Bootstrap resampling of 1000 replications was performed, and bootstrap values of >70 are given at branch nodes. Representative strains obtained from GenBank are identified by accession number, strain name, country of origin and year of isolation. Solid circles represent the HCV/HIV strains isolated in this study. Scale bars indicate nucleotide substitutions per site

Molecular characterization of HCV was performed as previously described [8]. Briefly, a fragment of the HCV NS5B region was amplified by reverse-transcriptase nested PCR. Amplification and sequencing of the NS5B fragment was successful in 22 samples (91.7 %), with the remaining 2 (8.3 %) having undetectable HCV RNA. A neighbor-joining phylogenetic tree was constructed with Kimura-2 parameter model using the MEGA6.0 software (http://www.megasoftware.net). Reference strains for HCV genotyping and worldwide NS5B sequences for phylogenetic analyses were retrieved from GenBank. Overall, the genotypic identity of HCV was diverse. A majority belonged to genotype 3a (n = 14; 63.6 %) followed by 1a (n = 4; 18.2 %); 6a (n = 2; 9.1 %); 1b (n = 1; 4.5 %); and 2a (n = 1; 4.5 %). Phylogenetic analysis suggested that there were a total of 8 circulating viruses, of which 2 showed significant clustering. One was a monophyletic HCV-3a cluster (bootstrap value >70 %), while the other was a HCV-6a pair (Fig. 2a). Members of the HCV-3a cluster were all diagnosed between 2013 and 2014. Despite the clustered HCV sequences, their corresponding HIV sequences did not show any genetic or temporal clustering. These samples contained HIV-1 of 2 subtypes: CRF01_AE (n = 5) and B (n = 8), as determined from *pol* gene sequences obtained for genotypic resistance testing [9] (Fig. 2b).

**Fig. 3:**
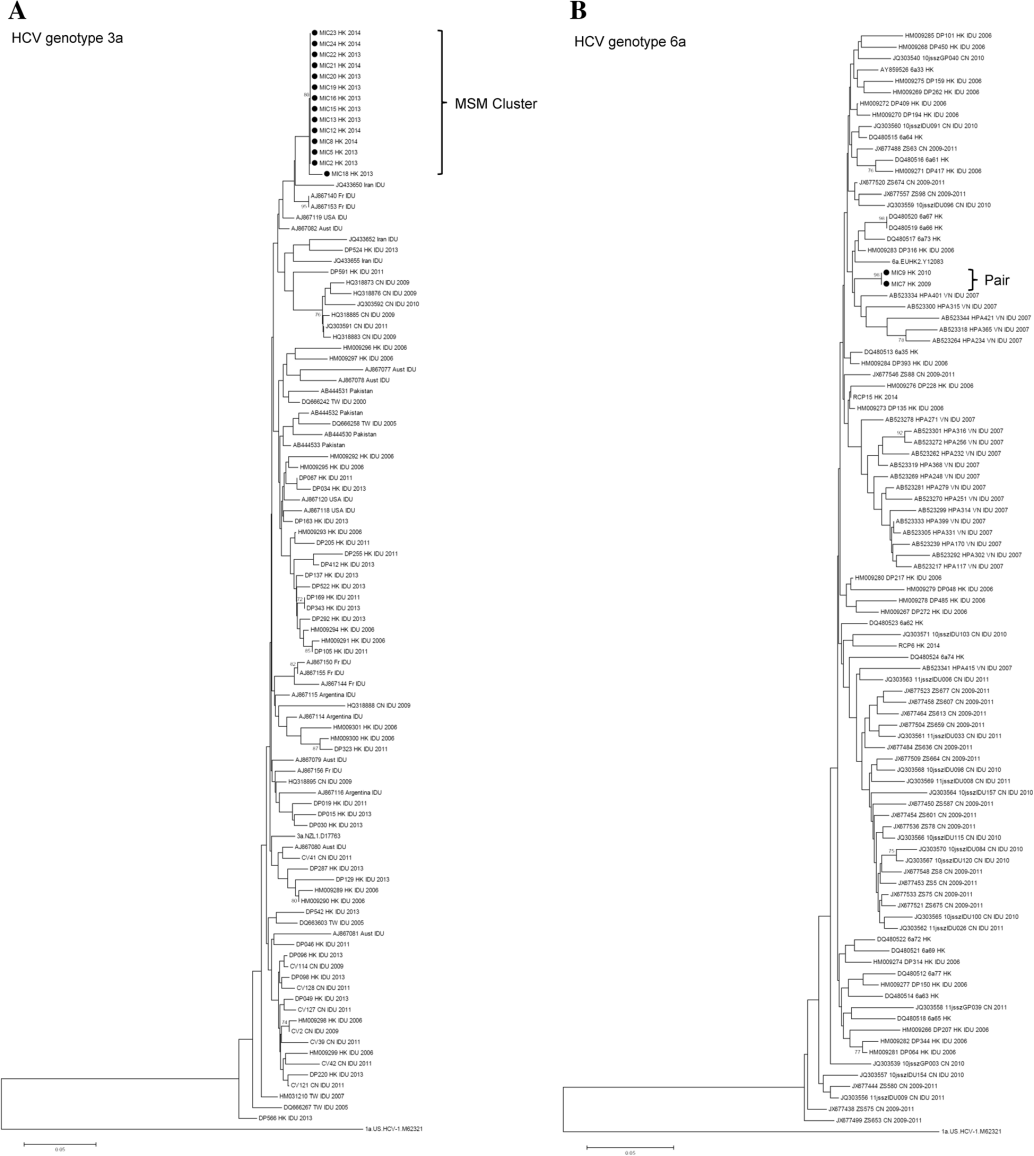
Phylogenetic analyses of nucleotide sequences of the partial NS5B gene of HCV-3a (**a**) and HCV-6a (**b**). The trees were constructed by the neighbor-joining method based on Kimura-2 parameter model. Bootstrap values of >70 are given at branch nodes. Representative strains obtained from GenBank are identified by accession number, strain name, country of origin and year of isolation. HCV-1a is included as outgroup. Solid circles represent the HCV strains isolated in this study. Scale bars indicate nucleotide substitutions per site

Figure 3a shows the results of phylogenetic analysis for the HCV genotype 3a samples. The HCV-3a sequences were distinctly different from those obtained from IDU in Hong Kong and Mainland China. The considerably shorter genetic distance between sequences within the HCV-3a cluster and their low genetic diversity, with diagnosis made within 2 years, suggested the occurrence of rapid virus spread in the HIV + ve MSM community, which might have resulted from a single source of recent introduction within Hong Kong. The origin of this cluster could not be further characterized as no similar sequences in Hong Kong could be found. HCV transmission might have arisen from the spillover of the virus from IDU to an MSM long time ago, as the sequences did not show any similarity with those in China, Southeast Asia or Australia. In Australia, genotype 3a actually constituted one of the most prevalent virus strains in acute HCV infections. Unlike the situation in Hong Kong, however, overlapping and coexistence of HCV-3a clusters between the IDU and HIV + ve MSM populations could be demonstrated in Australia [10].

Apart from the genotype 3a cluster, a homologous pair of near-identical genotype 6a sequences was identified, the diagnoses of which were made earlier in 2009 and 2010 before emergence of the HCV-3a cluster, with corresponding HIV diagnoses in 2006 and 2009. Further investigation revealed that the 2 persons were not known to one another. It is possible that the 2 near-identical virus strains formed part of a bigger but yet unidentified cluster, which could be circulating in HIV-ve MSM in Hong Kong. Alternatively, transmission might have ceased to occur after the diagnosis of the latest case in 2010. Phylogenetic analysis suggested that the HCV-6a sequence in the MSM pair was more similar to local IDU than those in Mainland China (Fig. 3b). Thus the virus might have been transmitted from a local MSM who injected drug, or through a sex partner who was an HCV infected IDU. The remaining 6 HCV viruses belonged to genotypes 1a, 1b and 2a. Globally, genotype 1 accounts for around 46 % of all HCV infections and is the most widely circulating genotype in Europe and North America [11]. Four of the viruses in our cohort belonged to this genotype, but all existed as singletons without evidence of clustering, and 2 were isolated from Caucasians. It is possible that these infections were acquired outside Hong Kong, or that local transmission has not occurred to a significant extent. The remaining HCV-2a infection, diagnosed in 2011, occurred in an HIV + ve MSM infected with HIV CRF07_BC, a signature HIV-1 subtype which originated in IDU in Yunnan with subsequent spread to Xinjiang and other provinces [12]. Apparently, local dissemination had not occurred for any of the non-3a HCV viruses after the diagnoses of these infections before 2012.

In conclusion, similar to the situation in Western countries, HCV has emerged as a sexually acquired infection in HIV + ve MSM in Hong Kong in the last decade. Phylogenetic investigations showed that 8 different HCV viruses were implicated in this new epidemic, of which one (HCV-3a) displayed significant genetic and temporal clustering. The HCV infections in HIV + ve MSM in Hong Kong did not form part of the reported international MSM-specific HCV transmission networks composing of HCV-1a and HCV-4d genotypes [13]. The discovery of a HCV-6a pair suggested that some of the infections might have arisen from local IDU. Evidently, the transmission of HIV and HCV in MSM in Hong Kong had differed both in onset time and their phylogenetic relationship. Apparently, HCV has spread in different MSM networks that became established after HIV infection. Instead of global dissemination in the MSM populations, HCV had probably come from local or nearby non-MSM sources, with multiple introductions at different time points. As sexual transmission of HCV is generally believed to be inefficient, its emergence in HIV + ve MSM populations could have resulted from an interlay of high risk sexual behaviors, host susceptibility and viral factors which have yet to be identified.

## Abbreviations

HCV: Hepatitis C virus
HIV + ve: HIV infected
IDU: Injection drug users
IQR: Interquartile range
MSM: Men who have sex with men
